# SIRT6 deficiency in endothelial cells exacerbates oxidative stress by enhancing HIF1α accumulation and H3K9 acetylation at the Ero1α promoter

**DOI:** 10.1002/ctm2.1377

**Published:** 2023-08-20

**Authors:** Zhenyang Guo, Xueting Yu, Shuang Zhao, Xin Zhong, Dong Huang, Runyang Feng, Peng Li, Zheyan Fang, Yiqing Hu, Zhentao Zhang, Mukaddas Abdurahman, Lei Huang, Yun Zhao, Xiangdong Wang, Junbo Ge, Hua Li

**Affiliations:** ^1^ Department of Cardiology, Zhongshan Hospital Shanghai Institute of Cardiovascular Diseases, Fudan University Shanghai China; ^2^ Department of Medical Examination Shanghai Xuhui District Central Hospital Shanghai China; ^3^ Department of Molecular Cell and Cancer Biology Program in Molecular Medicine University of Massachusetts Medical School MA USA; ^4^ School of Life Science and Technology ShanghaiTech University Shanghai China; ^5^ State Key Laboratory of Cell Biology Center for Excellence in Molecular Cell Science Chinese Academy of Sciences Shanghai Institute of Biochemistry and Cell Biology, University of Chinese Academy of Sciences Shanghai China; ^6^ Key Laboratory of Systems Health Science of Zhejiang Province School of Life Science Hangzhou Institute for Advanced Study, University of Chinese Academy of Sciences Hangzhou China; ^7^ Department of Pulmonary and Critical Care Medicine Zhongshan Hospital Shanghai Medical College Fudan University Shanghai China; ^8^ Department of Cardiology Zhongshan Hospital, Fudan University Shanghai China; ^9^ National Clinical Research Center for Interventional Medicine Shanghai China; ^10^ Shanghai Clinical Research Center for Interventional Medicine Shanghai China; ^11^ Key Laboratory of Viral Heart Diseases National Health Commission Shanghai China; ^12^ Key Laboratory of Viral Heart Diseases Chinese Academy of Medical Sciences Shanghai China

**Keywords:** endoplasmic reticulum stress, ischemia‒reperfusion injury, SIRT6

## Abstract

**Background:**

SIRT6, an important NAD^+^‐dependent protein, protects endothelial cells from inflammatory and oxidative stress injuries. However, the role of SIRT6 in cardiac microvascular endothelial cells (CMECs) under ischemia‒reperfusion injury (IRI) remains unclear.

**Methods:**

The HUVECs model of oxygen–glucose deprivation/reperfusion (OGD/R) was established to simulate the endothelial IRI in vitro. Endoplasmic reticulum oxidase 1 alpha (Ero1α) mRNA and protein levels in SIRT6‐overexpressing or SIRT6‐knockdown cells were measured by qPCR and Western blotting. The levels of H_2_O_2_ and mitochondrial reactive oxygen species (ROS) were detected to evaluate the status of oxidative stress. The effects of SIRT6 deficiency and Ero1α knockdown on cellular endoplasmic reticulum stress (ERS), inflammation, apoptosis and barrier function were detected by a series of molecular biological experiments and functional experiments in vitro. Chromatin immunoprecipitation, Western blotting, qPCR, and site‐specific mutation experiments were used to examine the underlying molecular mechanisms. Furthermore, endothelial cell‐specific Sirt6 knockout (ecSirt6^−/−^) mice were subjected to cardiac ischemia‒reperfusion surgery to investigate the effects of SIRT6 in CMECs in vivo.

**Results:**

The expression of Ero1α was significantly upregulated in SIRT6‐knockdown endothelial cells, and high Ero1α expression correlated with the accumulation of H_2_O_2_ and mitochondrial ROS. In addition, SIRT6 deficiency increased ERS, inflammation, apoptosis and endothelial permeability, and these effects could be significantly attenuated by Ero1α knockdown. The deacetylase catalytic activity of SIRT6 was important in regulating Ero1α expression and these biological processes. Mechanistically, SIRT6 inhibited the enrichment of HIF1α and p300 at the Ero1α promoter through deacetylating H3K9, thereby antagonizing HIF1α/p300‐mediated Ero1α expression. Compared with SIRT6‐wild‐type (SIRT6‐WT) cells, cells expressing the SIRT6‐H133Y‐mutant and SIRT6‐R65A‐mutant exhibited increased Ero1α expression. Furthermore, ecSirt6^−/−^ mice subjected to ischemia‒reperfusion surgery exhibited increased Ero1α expression and ERS in CMECs and worsened injuries to microvascular barrier function and cardiac function.

**Conclusions:**

Our results revealed an epigenetic mechanism associated with SIRT6 and Ero1α expression and highlighted the therapeutic potential of targeting the SIRT6‐HIF1α/p300‐Ero1α axis.

## Graphical abstract

Endoplasmic reticulum oxidase 1 alpha (Ero1α) expression is enhanced during ischemia‒reperfusion due to increased p300‐mediated H3K9Ac modification, resulting in increased endoplasmic reticulum stress (ERS) and endothelial dysfunctions. SIRT6 is responsible for inhibiting HIF1α/p300‐mediated Ero1α transcription by deacetylating H3K9 at the Ero1α promoter. Endothelial SIRT6 knockdown exacerbates ischemia‒reperfusion injury (IRI) in cardiac microvascular endothelial cells and cardiac impairment, highlighting its novel therapeutic potential for attenuating cardiac IRI.

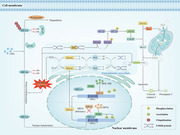

## 1 INTRODUCTION

Acute myocardial infarction (AMI) is still a leading cause of death and disability worldwide.^1^ However, although reperfusion therapies have been widely used in patients with AMI to continuously reduce ischemia‐induced myocardial damage, reperfusion itself tends to induce ischemia‒reperfusion injury (IRI) which paradoxically impairs the benefits of reperfusion therapy.^2^ The features of IRI include microembolization, capillary rupture and perfusion defects, leukocyte infiltration and sequential cardiomyocyte death.^3^ During this period, IRI in cardiac microvascular endothelial cells (CMECs) might occur much earlier than that in cardiomyocytes and with much greater severity. Moreover, the function of impaired CMECs has also been considered to largely determine the prognosis of these patients.^2^, ^4^ Therefore, it is critical to identify effective therapeutic targets for maintaining the functions of CMECs when they are exposed to IRI.

Previous studies have shown that endothelial IRI could induce endoplasmic reticulum stress (ERS) that reduces the ER protein‐folding capacity causing unfolded and misfolded proteins to accumulate du e to increased free radicals and the disruption of Ca^2+^ homeostasis and ER internal balance.^5^, ^6^ To alleviate these loads, the unfolded protein response (UPR) is generally activated to restore proteostasis.^7^ However, extensive or persistent UPR activation can make the UPR maladaptive, eventually leading to cell dysfunction or death. Thus, inhibiting maladaptive UPR might result in the amelioration of cellular dysfunction.^6^ The ER lumen is rich in a variety of chaperones, foldases and cofactors, and its oxidative environment collaboratively contributes to the formation of disulphide bonds and protein folding.^7^, ^8^ Endoplasmic reticulum oxidase 1 alpha (Ero1α), an important protein disulphide oxidase, is upregulated by the UPR to improve protein fold.^9^, ^10^

Recent studies have shown that SIRT6 is a negative regulator of ERS that alleviates ERS‐induced cellular dysfunction and apoptosis,^11^ and SIRT6 is an important sirtuin with unique and essential functions in maintaining endothelial homeostasis.^12^, ^13^, ^14^ In addition, genetic ablation of SIRT6 in ECs could significantly increase the sensitivity of cells to stimuli, such as hypoxia, inflammation and oxidative stress, and intensify ECs dysfunctions and apoptosis.^12^ In our previous work, SIRT6 was shown to alleviate ERS, reduce endothelial reactive oxygen species (ROS) levels and inhibit cellular apoptosis under oxidative stress.^15^ However, the molecular mechanism by which SIRT6 regulates endothelial ERS during cardiac IRI remains unknown. In this study, SIRT6 was shown to protect CMECs against ERS‐induced dysfunction and apoptosis under IRI by repressing HIF1α and deacetylating H3K9 to inhibit Ero1α expression.

## 2 RESULTS

### 2.1 SIRT6 negatively regulates the expression of Ero1α under OGD/R conditions

**FIGURE 1:**
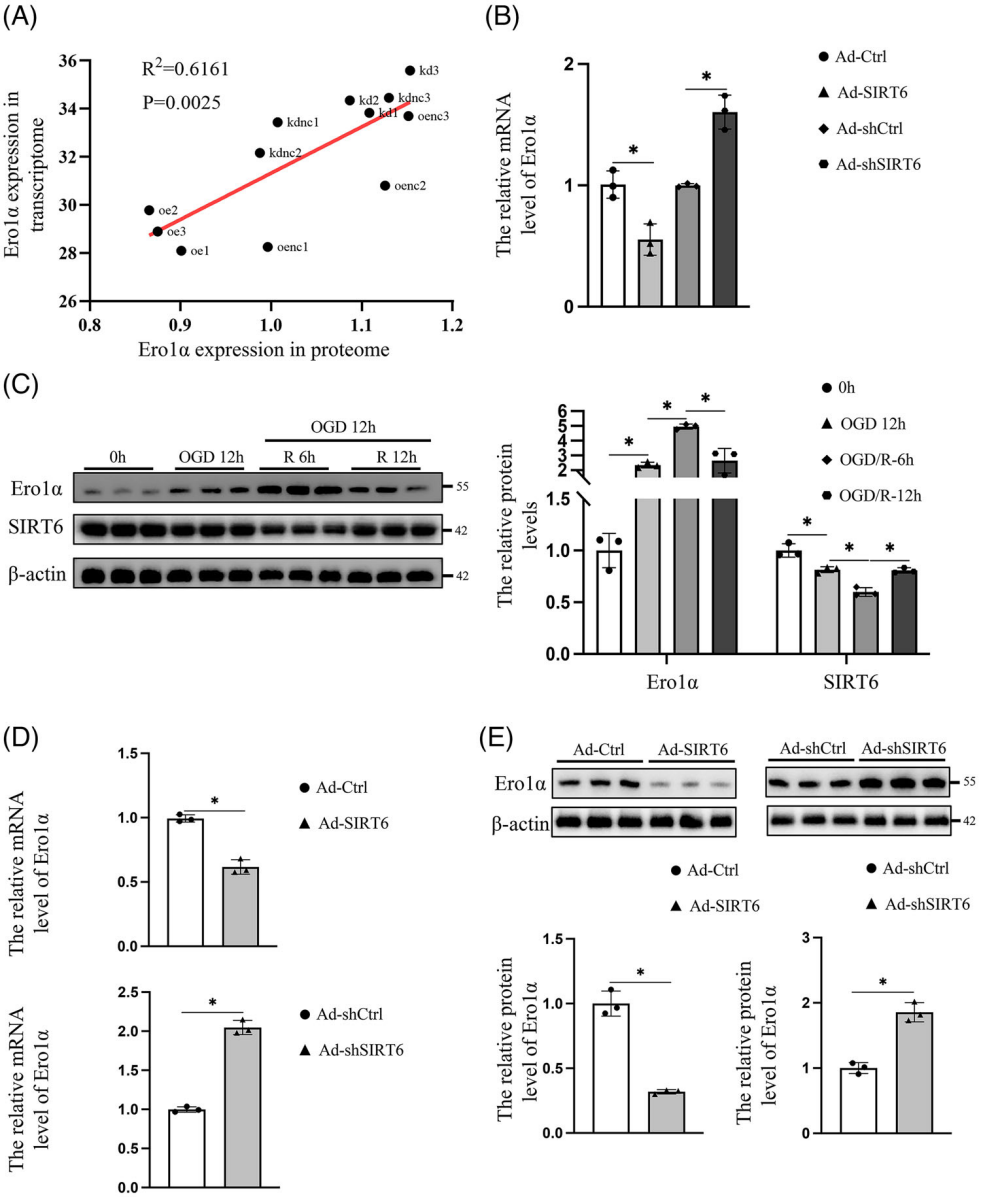
SIRT6 negatively regulates the expression of endoplasmic reticulum oxidase 1 alpha (Ero1α). (A) The correlation between Ero1α and SIRT6 in the proteome. (B) SIRT6 downregulated the expression of Ero1α in HUVECs exposed to 400 μM H_2_O_2_ for 4 h. (The original data are from Figure 2H of our previously published article.^15^ This is an open access article distributed under the terms of the Creative Commons CC BY license). (C) Changes in the protein expression Ero1α and SIRT6 in HUVECs at different times of OGD/R. (D) The mRNA and (E) protein levels of Ero1α in SIRT6‐overexpression or SIRT6‐knockdown HUVECs under OGD/R conditions. Ad‐Ctrl indicates the control of the SIRT6 overexpression; Ad‐SIRT6 indicates SIRT6 overexpression; Ad‐shCtrl indicates the control of the SIRT6 knockdown; and Ad‐shSIRT6 indicates SIRT6 knockdown. One‐way ANOVA followed by post hoc Tukey's test for C, and two‐tailed unpaired Student's *t*‐test for (D) and (E). ns means no significance; * means *p* < .05.

Ero1α gene and protein expression showed a strong negative linear correlation with SIRT6 in endothelial cells in our previous multiomics analysis (Figure 1A). In addition, our experimentally verified data also showed that SIRT6 negatively regulated the mRNA level of Ero1α in endothelial cells exposed to H_2_O_2_ (Figure 1B).^15^ To simulate vascular endothelial cell IRI in vitro, a glucose‐oxygen deprivation/reperfusion (OGD/R) injury cell model was established to determine the most appropriate conditions as the following experimental parameters.^16^, ^17^ During OGD, Ero1α expression was continuously upregulated, and its expression peaked at 12 h (Figure S1A). Then, during the cells were subjected to glucose–oxygen deprivation for 12 h before reperfusion, Ero1α expression was increased and peaked at 6h of reperfusion, followed by a gradual decline (Figure S1B). More importantly, the protein level of SIRT6 reached its lowest level after 12 h of OGD followed by 6 h of reperfusion (Figure 1C). Therefore, HUVECs subjected to 12 h of OGD followed by 6 h of reperfusion were selected as our following study subjects. To revalidate the intermolecular relationship between SIRT6 and Ero1α under OGD/R conditions, SIRT6‐overexpression or SIRT6‐knockdown HUVECs were generated (Figure S1C). The results indicated that SIRT6 overexpression significantly reduced mRNA and protein levels of Ero1α, while SIRT6 knockdown had the opposite effect (Figure 1D,E). Collectively, OGD/R could stimulate the expression of Ero1α, and that SIRT6 can reduce Ero1α expression under this condition.

### 2.2 SIRT6 deficiency increases endothelial dysfunction, apoptosis and inflammation under OGD/R conditions

**FIGURE 2:**
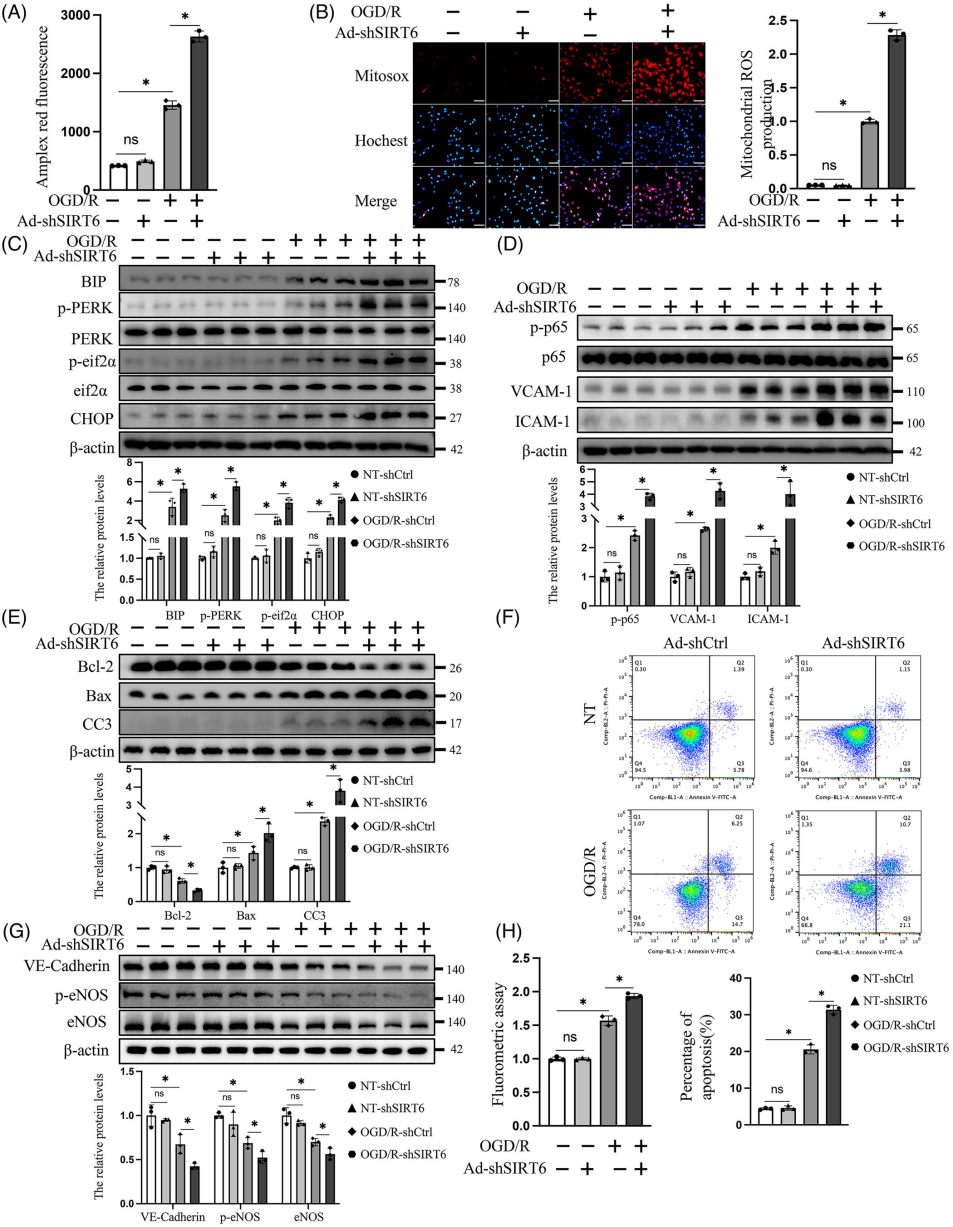
SIRT6 deficiency exacerbates endothelial dysfunction under OGD/R conditions. (A) H_2_O_2_ levels were measured by using the Amplex red hydrogen peroxide/peroxidase assay. (B) Representative (*left*) and statistical analysis (*right*) of mitochondrial reactive oxygen species levels were determined by using mitochondrial superoxide indicators in HUVECs. Scale bars = 40 μm. (C) Endoplasmic reticulum stress, (D) inflammation and (E) apoptosis markers were detected by Western blotting. (F) Flow cytometry was used to evaluate the effect of SIRT6 on HUVEC apoptosis (*upper*). Quantification of the cell apoptosis is shown on the *lower*. (G) Representative Western blots and statistical analysis of VE‐cadherin, p‐eNOS and eNOS in HUVECs. (H) Quantitative assay of endothelial permeability with the fluorescence intensity ratio of the lower chamber versus upper chamber. NT indicates the control of OGD/R treatment group; Ad‐shCtrl indicates the control of SIRT6 knockdown; and Ad‐shSIRT6 indicates SIRT6 knockdown. One‐way ANOVA followed by post hoc Tukey's test was used. ns means no significance; * means *p* < .05.

Previous studies showed that Ero1α was an important contributor to H_2_O_2_ generation in the ER.^9^ In this study, OGD/R treatment could significantly increase endothelial H_2_O_2_ accumulation and this effect was exacerbated by SIRT6 knockdown (Figure 2A). In addition, Ero1α may affect redox signalling and calcium flux in mitochondria through diffusible H_2_O_2_ to disrupt the cellular ROS homeostasis.^9^ Therefore, the ROS levels in mitochondria were detected by using mitochondrial superoxide indicators. These results revealed that mitochondrial ROS levels were significantly increased under OGD/R conditions, and this effect was exacerbated by SIRT6 knockdown (Figure 2B). With continuous accumulation of ROS, the overactivation of ERS and maladaptive UPR can occur. In HUVECs, increased levels of the unfolded protein sensor BIP and hyperactivation of PERK‐eif2α signalling pathway were observed in SIRT6‐knockdown group under OGD/R conditions (Figure 2C). Notably, increased expression of CHOP suggested that SIRT6 deficiency could accelerate the maladaptive UPR (Figure 2C). In addition, hyperactivation of NF‐κB signalling and inflammation reactions were observed in SIRT6‐deficient HUVECs (Figure 2D).

Continuous maladaptive UPR and excessive inflammatory reactions could lead to cellular apoptosis. Under OGD/R conditions, SIRT6 deficiency resulted in increased levels of the proapoptotic proteins Bax and cleaved caspase‐3 (CC3), while the level of the antiapoptotic protein Bcl‐2 was reduced (Figure 2E). Similarly, increase in apoptosis caused by SIRT6 knockdown was also verified by flow cytometry (Figure 2F). Additionally, SIRT6 deficiency exacerbated endothelial dysfunction, as indicated by reduced protein levels of VE‐cadherin, p‐eNOS and eNOS in SIRT6‐knockdown HUVECs under OGD/R conditions (Figure 2G). Subsequently, transwell model was employed to detect the endothelial permeability^18^ (Figure S1D). The results showed that OGD/R increased endothelial permeability, while SIRT6 knockdown significantly increased endothelial permeability, with more dextran‐FITC in the lower chamber (Figure 2H).

### 2.3 Ero1α knockdown reverses OGD/R‐induced endothelial injury in SIRT6‐deficient cells

**FIGURE 3:**
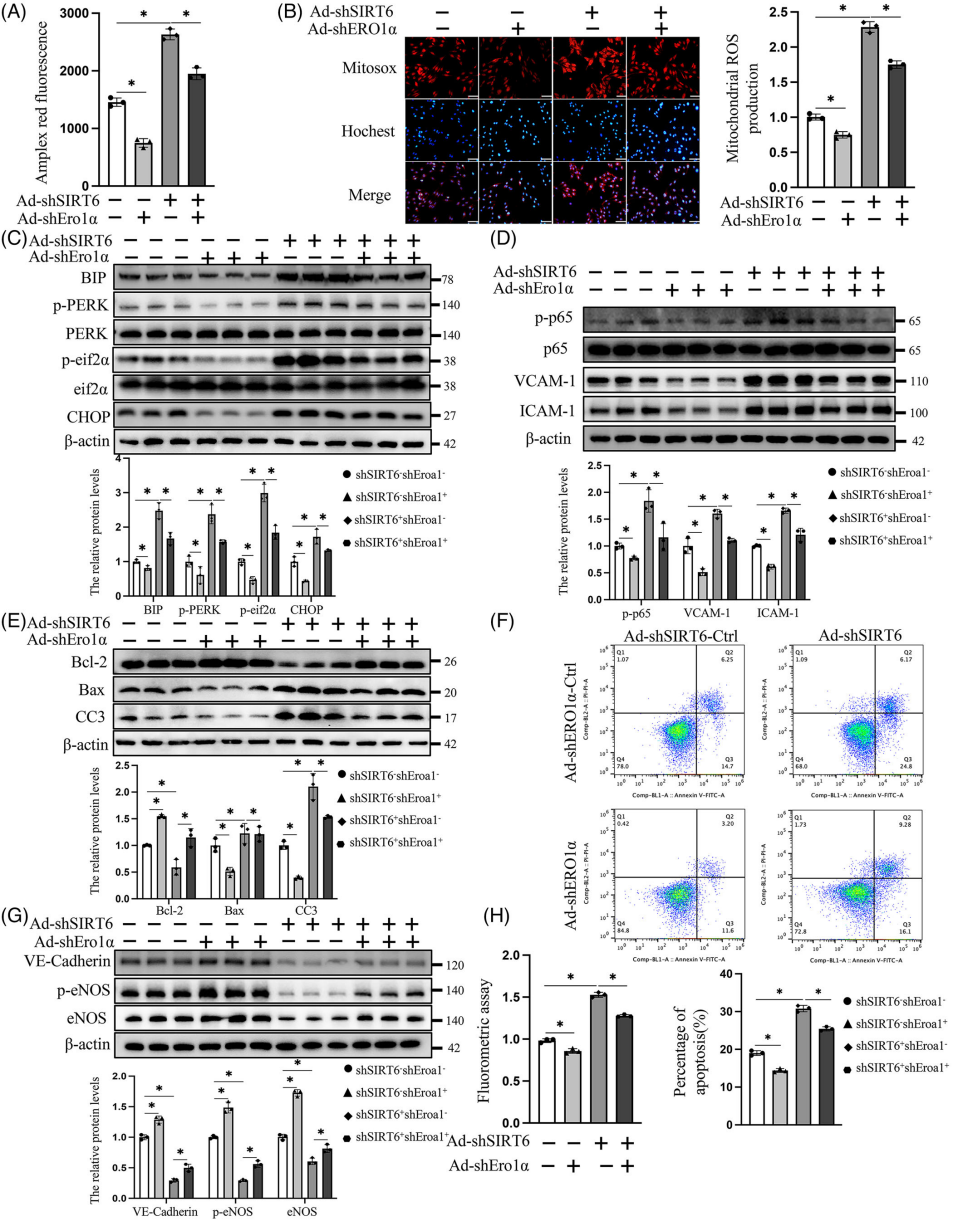
Endoplasmic reticulum oxidase 1 alpha (Ero1α) knockdown reverses OGD/R‐induced endothelial injury in SIRT6‐deficient cells. (A) H_2_O_2_ levels in HUVECs were measured using the Amplex red hydrogen peroxide/peroxidase assay. (B) Mitochondrial superoxide levels were determined via using mitochondrial superoxide indicators in HUVECs under OGD/R conditions. Scale bars = 40 μm. Western blot analysis and quantification of endoplasmic reticulum stress (C), inflammation (D) and apoptosis (E) markers in HUVECs under OGD/R. (F) Flow cytometric analysis (*upper*) and quantification (*lower*) of HUVEC apoptosis. (G) Western blotting and statistical analysis of the protein expression of VE‐cadherin and eNOS in HUVECs under OGD/R. (H) Endothelial permeability was measured in a transwell model. shSIRT6^−^ indicates the control of SIRT6 knockdown; Ad‐shSIRT6 or shSIRT6^+^ indicates the SIRT6‐knockdown group; shEro1α^−^ indicates the control of Ero1α knockdown; and Ad‐shEro1α or shEro1α^+^ indicates the Ero1α knockdown. One‐way ANOVA followed by post hoc Tukey's test was used. ns means no significance; * means *p* <  .05.

To investigate whether SIRT6 deficiency‐induced endothelial injury could be ameliorated by Ero1α depletion, Adv‐mediated Ero1α‐knockdown HUVECs were generated (Figure S2A). First, Ero1α knockdown significantly decreased the accumulation of H_2_O_2_ mitochondrial ROS in SIRT6‐knockdown HUVECs and their corresponding controls under OGD/R conditions (Figure 3A,B). Second, related biomarkers of ERS were examined as previously described to evaluate the effect of Ero1α knockdown on the activation of ERS. We found that the Ero1α knockdown significantly inhibited SIRT6 deficiency‐induced hyperactivation of ERS under OGD/R conditions, as indicated by decreased expression of BIP, p‐PERK and p‐eif2α and CHOP (Figure 3C). Similarly, Ero1α knockdown also significantly suppressed the endothelial inflammatory reaction in SIRT6‐deficient HUVECs, as evidenced by the inhibition of NF‐κB signalling and decreased expression of ICAM‐1 and VCAM‐1(Figure 3D). Third, the increase in apoptosis in SIRT6‐knockdown HUVECs was rescued by Ero1α knockdown, which was evaluated by caspase3 activity analysis (Figure 3E) and flow cytometry analysis (Figure 3F). Additionally, Ero1α silencing could restore the protein levels of VE‐cadherin, p‐eNOS and eNOS in SIRT6‐deficient HUVECs under OGD/R conditions (Figure 3G), which suggested that inhibiting Ero1α could substantially ameliorate OGD/R‐induced endothelial dysfunction in the absence of SIRT6. Based on the above data, the effects of Ero1α knockdown on endothelial permeability was assessed. Compared with the corresponding group, Ero1α knockdown significantly reduced the abnormal cellular permeability in the SIRT6‐knockdown group (Figure 3H). In summary, these results suggested that Ero1α knockdown effectively rescued endothelial dysfunction and inhibited endothelial apoptosis and inflammation in SIRT6 deficiency under OGD/R conditions.

### 2.4 SIRT6 inhibits Ero1α by suppressing HIF1α accumulation and H3K9Ac in its promotor

**FIGURE 4:**
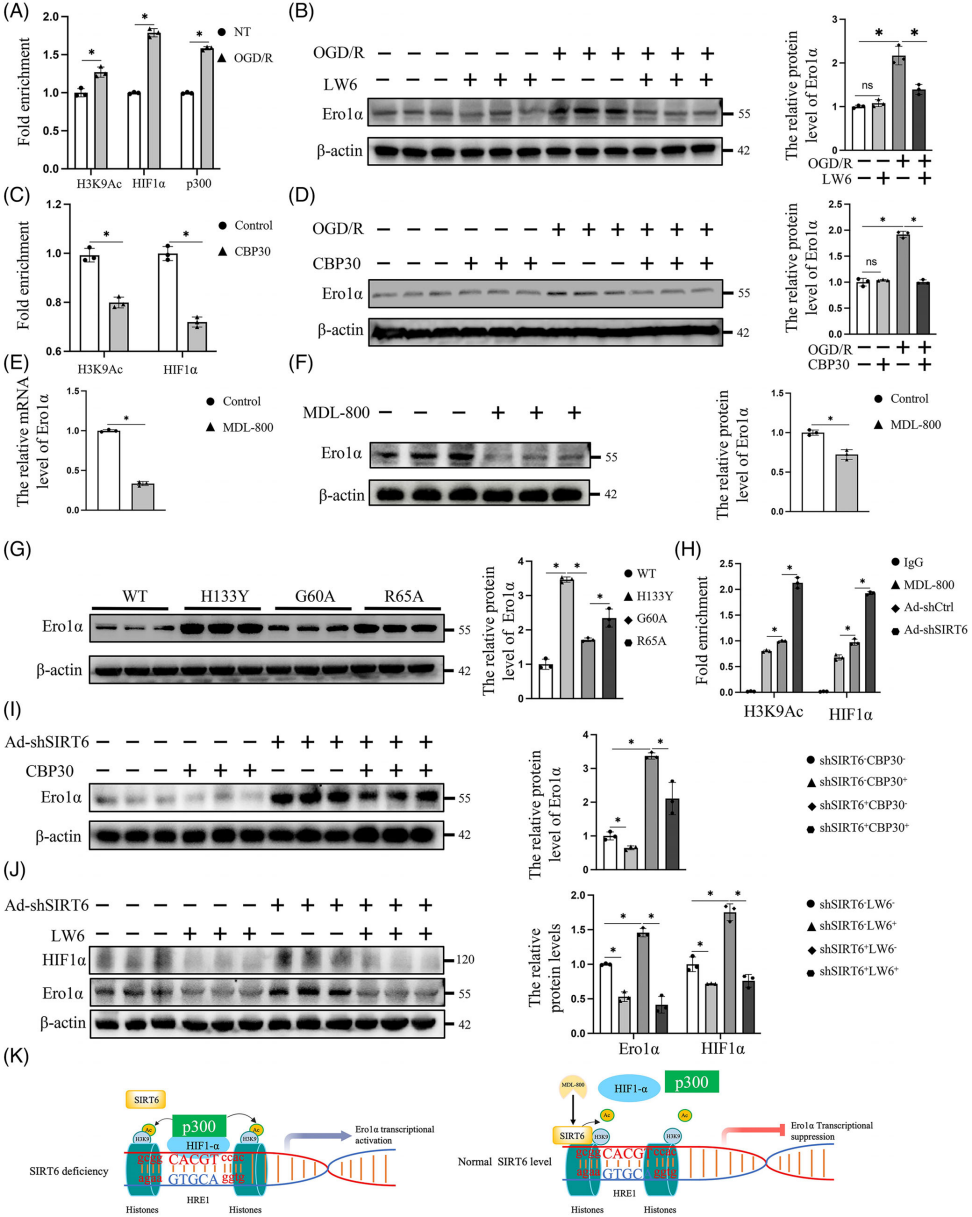
SIRT6 inhibits endoplasmic reticulum oxidase 1 alpha (Ero1α) by suppressing HIF1α accumulation and H3K9Ac in its promotor. (A) Chromatin immunoprecipitation (ChIP) assays were performed to detect the levels of H3K9Ac, HIF1α and p300 at the Ero1α promoter in HUVECs. IgG was used as negative control. (B) The protein level of Ero1α was detected by Western blotting. (C) ChIP assays were performed on HUVECs treated with PBS or CBP30, with antibodies against H3K9Ac and HIF1α. (D) The protein level of Ero1α was detected by Western blotting. The effect of MDL‐800 on the expression of Ero1α was determined by qPCR (E) and Western blotting (F) in HUVECs under OGD/R treatment. (G) The protein level of Ero1α was detected in 293T cells under OGD/R treatment by Western blotting. (H) The levels of H3K9Ac and HIF1α at the Ero1α promoter were detected by ChIP analysis of HUVECs under OGD/R conditions. (I,J) Under OGD/R conditions, the effects of CBP30 and LW6 on the protein level of Ero1α were detected by Western blotting in SIRT6‐knockdown HUVECs and the corresponding controls. (K) Schematic model showing the enrichment of the HIF1α/p300 complex at the Ero1α promoter to upregulate the expression of Ero1α under OGD/R conditions. Under normal conditions, SIRT6 can suppress the recruitment of HIF1α and acetylation of H3K9 at the Ero1α promoter to reduce the expression of Ero1α. shSIRT6^−^ indicates the control of SIRT6 knockdown; Ad‐shSIRT6 or shSIRT6^+^ indicates the SIRT6 knockdown; LW6^−^ indicates the control of LW6 treatment; LW6^+^ indicates the LW6 treatment; CBP30^−^ indicates the control of CBP30 treatment; and CBP30^+^ indicates the CBP30 treatment. Two‐tailed Student's unpaired *t*‐test for (A), (C), (E) and (F) and one‐way ANOVA followed by post hoc Tukey's test for (B), (D), (G), (H), (I) and (J). ns means no significance; * means *p* < 0.05.

A previous study revealed that HIF1α accumulated in HRE1 (−191/−184 nt) of the Ero1α promoter and triggered Ero1α expression under Hcy stimulation.^19^ As a transcription factor, HIF1α can generally form a complex with the cotranscription factor p300 to bind to this hypoxia‐response element (HRE) and then trigger downstream responses.^20^, ^21^ Therefore, in this study, we investigated whether Ero1α expression was regulated by the HIF1α/p300 transcriptional complex under OGD/R conditions. The chromatin immunoprecipitation (ChIP) assay showed that the binding of HIF1α and p300 to HRE1 of the Ero1α promoter was significantly promoted under OGD/R conditions (Figure 4A). In addition, although there was no significant inhibitory effect at the Ero1α expression under normal conditions, LW6 (a selective inhibitor for decreasing stability of the HIF1α protein^22^) was obviously effective in reducing the protein level of Ero1α in HUVECs under OGD/R conditions (Figure 4B). The histone acetyltransferase p300 is responsible for histone acetylation, facilitating nucleosome rearrangement and DNA binding by RNA‐polymerase II and transcription factors.^23^ Under OGD/R conditions, H3K9 acetylation (H3K9Ac) at the Ero1α promoter was also significantly increased (Figure 4A). Based on these findings, the effects of p300 modulation of H3K9Ac and HIF1α at the Ero1α promoter were further examined, and the results showed that CBP30 (a selective inhibitor for inhibiting p300 activity^24^) significantly decreased the accumulation of H3K9Ac and HIF1α at the Ero1α promoter (Figure 4C). In addition, the protein level of Ero1α was also downregulated by CBP30 in HUVECs under OGD/R conditions but not in normal condition (Figure 4D). The above data indicated that the expression of Ero1α was regulated in an HIF1α/p300 transcriptional complex dependent manner under OGD/R, and p300 was responsible for both H3K9Ac accumulation and binding of HIF1α to the Ero1α promoter.

A published paper showed that SIRT6 was a corepressor of HIF1α and suppressed the expression of HIF1α target genes by deacetylating H3K9 at their promoters.^25^ Based on these findings, we then investigated whether SIRT6 modulated Ero1α, the target gene of HIF1α, through deacetylating H3K9. We found that activating deacetylation activity of SIRT6 by MDL‐800 (a novel SIRT6 deacetylase activity activator^26^) substantially inhibited both the expression of Ero1α mRNA and that of its protein (Figure 4E,F). To further examine the relationship between SIRT6 enzymatic activity and Ero1α expression, SIRT6‐knockout (SIRT6‐KO) 293T cells were generated (Figure S2B). These cells were then transfected with lentivirus encoding wild‐type SIRT6 or different SIRT6 mutants. Previous studies reported that H133Y lacks both deacetylase activity and mono‐ADP‐ribosyl transferase activity. R65A impairs deacetylase activity, but mono‐ADP‐ribosyl transferase activity is retained, while G60A has the opposite effect.^12^ Compared with G60A, cells expressing SIRT6‐H133Y or SIRT6‐R65A exhibited higher protein level of Ero1α under OGD/R conditions (Figure 4G), suggesting that the deacetylase activity but not mono‐ADP‐ribosyl transferase activity of SIRT6 is responsible for the inhibition of Ero1α expression. Additionally, ChIP assay showed that SIRT6 deficiency led to increased accumulation of HIFα and H3K9Ac levels at the promoter of Ero1α under OGD/R, but activation of SIRT6 deacetylation activity by MDL‐800 had the opposite effect (Figure 4H, Figure S2C). In addition, compared with that in the control group, inhibiting p300 activity or depleting of HIF1α effectively reversed the change in the expression of Ero1α in SIRT6‐knockdown HUVECs under OGD/R (Figure 4I,J).

In summary, OGD/R treatment promoted Ero1α expression in an HIF1α/p300‐dependent manner. SIRT6 functioned as a corepressor of HIF1α to inhibit the enrichment of HIF1α in the Ero1α promoter through antagonizing the p300‐induced H3K9Ac to suppress the expression of Ero1α (Figure 4K).

### 2.5 SIRT6 attenuated the IRI of CMECs in vivo

**FIGURE 5:**
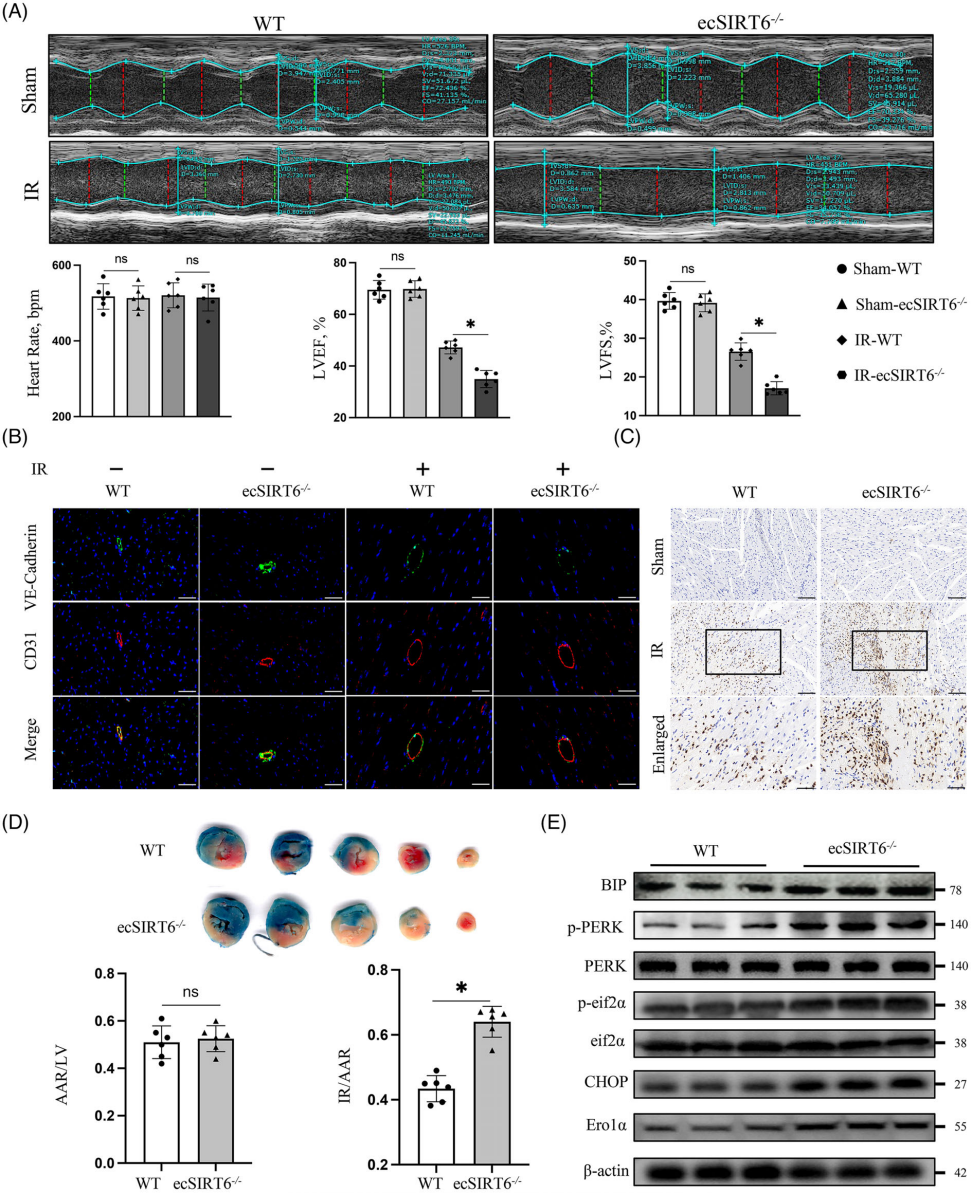
Sirt6 knockout exacerbates ischemia‒reperfusion injury of cardiac microvascular endothelial cells (CMECs) in vivo. (A) (*Upper*) Representative cardiac M‐mode echocardiograms were performed after 6 h of cardiac IR surgery or sham operation in ecSirt6^−/−^ mice and WT mice. (*Lower*) Heart rates and cardiac systolic functions (LVEF, LVFS) were quantified. *N* = 6/group. (B) Endothelial barrier integrity was assessed by co‐staining of VE‐cadherin and CD31. Scale bars = 50 μm. *N* = 6/group. (C) Representative images showing the Ly6G^+^ neutrophil infiltration in the myocardial tissues. Scale bars = 100 or 50 μm (enlarged groups). *N* = 6/group. (D) (*Upper*) Representative images showing TTC stained transverse sections of hearts perfused by Evans blue in each group. (*Lower*) AAR/LV and IA/AAR were quantified to evaluate the cardiac infarct size. The ratio of infarct area to area at risk (IA/AAR) indicates the infract size. *N* = 6/group. (E) Western blotting was used to determine the protein expression of endoplasmic reticulum stress markers and endoplasmic reticulum oxidase 1 alpha in mouse CMECs from ecSirt6^−/−^ mice and WT mice after cardiac IR surgery. *N* = 6/group. One‐way ANOVA followed by post hoc Tukey's test for (A), and two‐tailed Student's unpaired *t*‐test for (D) and (E). ns means no significance; * means *p* < 0.05.

In order to investigate the effects of endothelial SIRT6 deficiency in IRI of CMECs in vivo, endothelial cells‐specific Sirt6 knockout (ecSirt6^−/−^) mice were created by using the Cre‐LoxP recombination system (Figure S3A). Wild‐type (WT) and ecSirt6^−/−^ mice were identified by adopting mouse tail genotyping and Western blot analysis of mouse coronary endothelial cells (Figure S3B,C). In addition, to test the therapeutic potential of endothelial SIRT6 in the cardiac IR, we induced Sirt6 overexpression in endothelial cells of ecSirt6^−/−^ mice by intravenous injection of adeno‐associated virus‐9 encoding Sirt6 (AAV‐Sirt6‐OE). At 4 weeks post‐injection, a ∼twofold increase in Sirt6 expression in CMECs was observed (Figure S3D). Compared with mice in the control group, ecSirt6^−/−^mice exhibited more serious damage of cardiac functions in the context of cardiac IRI, including the deterioration of left LVEF and LVFS (Figure 5A). However, overexpression of SIRT6 in ecSirt6^−/−^mice yielded improved cardiac functions after cardiac ischemia reperfusion (Figure S4A).

VE‐cadherin, a junctional protein responsible for maintaining the microvascular barrier, was impaired by IRI, which caused inflammatory cell migration and infiltration.^27^ In ecSirt6^−/−^ mice, the continuous VE‐cadherin fluorescence was disrupted more seriously by cardiac IRI than in WT mice, while endothelial Sirt6‐overexpression mice presented more continuous VE‐cadherin fluorescence (Figure 5B, Figure S4B). Subsequently, the collapse of vascular endothelial barrier was followed by more infiltration of neutrophils (marked by Ly6G^+^) in the damaged myocardial tissues in ecSirt6^−/−^mice, which far exceed WT mice (Figure 5C). This phenotype was significantly rescued by endothelial Sirt6 overexpression (Figure S4C). In addition, increased infarct size, as evaluated by the ratio of IA to AAR, occurred in ecSirt6^−/−^ mice (Figure 5D). Conversely, Sirt6 overexpression in endothelial cells resulted in a drastic reduction in cardiac infarct size (Figure S4D). Furthermore, to examine the effect of Sirt6 on ERS activation in vivo, CMECs were isolated from mice that underwent cardiac IR surgery. The quality control of mice CMECs was accomplished by flow cytometry analysis and immunofluorescence for CD31 positivity (Figure S3E,F). We found that endothelial Sirt6 knockout resulted in higher protein expression of Ero1α and overactivation of ERS, but Sirt6 overexpression had the opposite effect (Figure 5E, Figures S3G and S4E).

### 2.6 Ero1α knockdown mitigates IRI of CMECs in ecSirt6^−/−^ mice in vivo

To clarify the effects of Ero1α in IRI of CMECs in vivo, endothelial‐specific Ero1α‐knockdown mouse model mediated by the AAV9 encoding Ero1α shRNA 9 (AAV9‐shEro1α) was established (Figure S5A). The knockdown efficiency and specificity of endothelial Ero1 in vivo were confirmed by Western blot. The protein level of Ero1α in isolated CMECs from AAV9‐shEro1α mice was manifested a significant decrease compared to that of controls (Figure S5B), but there was no significant difference in cardiomyocytes (Figure S5C).

**FIGURE 6:**
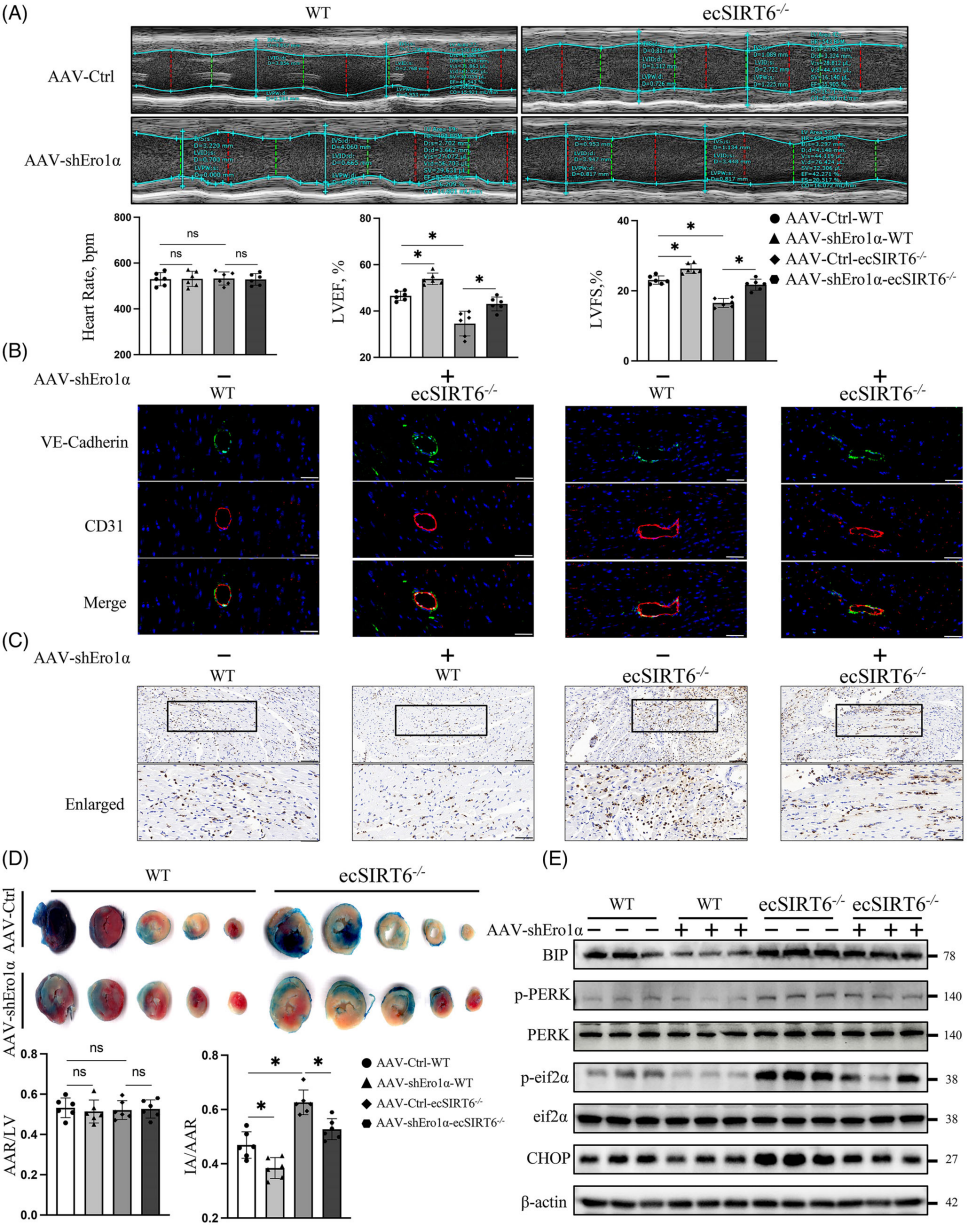
Endoplasmic reticulum oxidase 1 alpha (Ero1α) knockdown mitigates ischemia‒reperfusion injury of cardiac microvascular endothelial cells (CMECs) in ecSirt6^−/−^ mice in vivo. (A) Representative cardiac M‐mode echocardiograms and cardiac systolic functions (LVEF, LVFS) were quantified. *N* = 6/group. (B) Co‐staining of VE‐cadherin and CD31 was performed to characterize endothelial barrier integrity. Scale bars = 50 μm. *N* = 6/group. (C) Representative images showing the Ly6G^+^ neutrophil infiltration in myocardial tissues. Scale bars = 100 or 50 μm (enlarged groups). *N* = 6/group. (D) The infarcted area was quantified by TTC staining. *N* = 6/group. (E) Endoplasmic reticulum stress markers were detected by Western blotting in isolated mouse CMECs from ecSirt6^−/−^ mice and WT mice after cardiac IR surgery. *N* = 6/group. AAV‐Ctrl indicates the control of AAV‐mediated endothelial Ero1α knockdown; AAV‐shEro1α indicates the AAV‐mediated endothelial Ero1α knockdown. One‐way ANOVA followed by post hoc Tukey's test was used. ns means no significance; * means *p* < 0.05.

We found that endothelial‐specific Ero1α knockdown could markedly rescue cardiac functions such as LVEF and LVFS in ecSirt6^−/−^ mice (Figure 6A). In addition, the endothelial Ero1α knockdown also improved VE‐cadherin continuity and inhibited the neutrophils (Ly6G^+^) migration into the damaged myocardial tissue (Figure 6B,C). Accordingly, AAV9‐Ero1α‐shRNA‐infected ecSirt6^−/−^ mice exhibited a lower ratio of IA/AAR (Figure 6D), which was consistent with the previous results that Ero1α knockdown reduced cell injuries. To investigate the effects of SIRT6 knockout and Ero1α knockdown on ROS homeostasis during endothelial IR, CMECs were isolated and subjected to a mitochondrial superoxide assay. The results showed that SIRT6 depletion exacerbated ROS production, and specific endothelial Ero1α knockdown rescued the upregulation of ROS production in vivo (Figure S6A). Regarding ERS activation, endothelial Ero1α knockdown effectively suppressed the increase in the protein expression of BIP, p‐PERK, p‐eif2α and CHOP in ecSirt6^−/−^ mice subjected to cardiac IR (Figure 6E, Figure S6B). In addition, hyperactivation of p‐p65 and the endothelial inflammatory response were also suppressed by Ero1α knockdown (Figure S6C). Collectively, these above data indicated that endothelial Ero1α knockdown could reverse IR‐induced ERS and microvascular damage in ecSirt6^−/−^ mice.

## 3 DISCUSSION

Timely reperfusion remedy is one of most effective measure to rescue ischemic cardiac tissue, but this treatment unexpectedly damages the cardiac endothelial cells, playing a decisive role in the pathogenesis of microvascular and cardiomyocyte complications.^3^ Furthermore, microvascular bed injuries impose additional myocardial damage risk and increase the 30‐day mortality rate.^28^, ^29^ A previous study showed that SIRT6 silencing in brain microvascular endothelial cells significantly aggravated the endothelial apoptosis and blood–brain barrier damage in mice exposed to IR, and decreased infarct size and neurological deficit.^13^ Considering the crucial role of microvasculature and endothelial cells in cardiac function in AMI patients after reperfusion, we investigated the function of endothelial SIRT6 in cardiac IRI by using human primary cells and animal models.

In this study, the protective role and molecular mechanism of SIRT6 in ECs after cardiac IRI was first investigated. We found that SIRT6 inhibited HIF1α transcriptional activity by antagonizing p300 acetylation of H3K9 at the Ero1α promoter, thereby suppressing Ero1α expression and augmentation of ERS‐induced endothelial dysfunction. These results suggested SIRT6‐HIF1α/p300‐Ero1α axis may be a promising novel therapeutic target. Data as followed in this study substantiated our conclusions: (1) SIRT6 expression was reduced but Ero1α expression was increased in primary HUVECs exposed to OGD/R in vitro; (2) SIRT6‐knockdown resulted in increased expression of Ero1α and activation of ERS, and impaired the endothelial functions in HUVECs exposed to OGD/R in vitro; (3) endothelial‐specific Sirt6 genetic deletion exacerbated cardiac microvascular damage and cardiac function impairment by increasing Ero1α and hyperactivation of ERS; (4) In vivo Sirt6 overexpression in CMECs attenuated IR‐induced cardiac microvascular damage and improves cardiac outcome; (5) Ero1α knockdown rescued the endothelial dysfunction and cardiac function impairment in vitro and in vivo.

SIRT6 was previously reported to promote ROS‐induced ERS through high activation of PERK/eIF2α signalling in SIRT6‐overexpression papillary thyroid cancer cells.^30^ However, SIRT6 was also shown to ameliorate hepatic ERS mainly by blocking XBP1s and less via the PERK/eIF2α pathway.^11^ In this study, SIRT6 deficiency resulted in hyperactivation of the PERK/eIF2α pathway because of increased expression of Ero1α. Cell‐type specificities or different external stimuli may have played different roles. Although activation of the PERK/eIF2α pathway helped cell adaptation in response to ERS, it also promoted the expression of the proapoptotic factor CHOP to induce apoptosis when cells were under persistent ERS.^31^ In addition, MDL‐800, the small molecule allosteric activator of SIRT6, inhibited the HIF1α occupation within the Ero1α promoter and Ero1α expression, suggesting its therapeutic potential for endothelial IRI. Consistently, the results showed that knockdown or genetic deletion of SIRT6 could promote cell apoptosis induced by excessive ERS. In CMECs isolated from ecSirt6^−/−^ mice that underwent cardiac IR surgery, increased activation of PERK/eIF2α signalling was similarly observed. Collectively, these above findings suggest that SIRT6 deficiency activates the PERK/eIF2α signalling pathway and exacerbates the endothelial dysfunction and cell apoptosis under ERS.

In the transcriptional regulation of gene expression, OGD/R‐induced Ero1α expression resulted from the combined effects of HIF1α and p300 in this study. HIF1α, a known vascular transcription factor, is involved in endothelial cell dysfunction, angiogenesis and inflammation.^32^, ^33^ In nonhypoxic conditions, HIF1α is hydroxylated, and subsequently recognized by the von Hippel–Lindau (VHL) ubiquitin ligase, which marks HIF1α for subsequent proteasome degradation.^32^ When oxygen is unavailable, the process of HIF1α degradation is blocked, and HIF1α translocates to the nucleus and binds to the HRE of target genes.^32^ P300 regulates the acetylation status of lysine residues of histones at HIF‐1 target genes, relaxing the chromatin structure and promoting HIF‐1‐dependent gene transcription.^23^, ^34^, ^35^ A previous study revealed that SIRT6 suppressed HIF1α transcriptional activity by directly deacetylating H3K9 at HIF1α target gene promoters and increased protein synthesis and stability of HIF1α were observed in SIRT6‐deficient cells.^25^, ^36^ The above results suggested that SIRT6 could downregulate the transcription of HIF1α target genes by influencing HIF1α transcriptional activity and protein level. On the other hand, Yang et al. observed that SIRT6 prevented HIF1α from degradation by reducing the ubiquitination of HIF1α, thus promoting HIF1α accumulation and the expression of its target gene VEGF under normoxia and hypoxia in HUVECs.^37^ Consistent with the first view, the data of our experiment indicated that SIRT6 competed with p300 to reduce the recruitment of HIF1α to the Ero1α promoter by maintaining histones in a hypoacetylated state. These results suggested that SIRT6 regulated the expression of Ero1α by inhibiting HIF1α transcriptional activity through deacetylating H3K9 under OGD/R conditions.

Ero1α is positively regulated by ERS and participates in a pivotal pathway that catalyses oxidative protein folding.^38^, ^39^ Overexpression of Ero1α disrupts ER redox homeostasis because of the excessive production of H_2_O_2_ during assisting protein folding.^19^ By increasing Ca^2+^ efflux from the ER and mitochondrial superoxide formation, Ero1α can trigger ERS and apoptosis.^40^, ^41^ Therefore, there seems to be a positive loop in which ER stress induces Ero1α expression and Ero1α promotes H_2_O_2_ accumulation and mitochondrial ROS formation, thereby amplifying ER stress and causing cellular dysfunction and apoptosis.^42^ In this study, high expression of Ero1α increases the levels of cellular H_2_O_2_ and mitochondrial ROS, ERS‐related apoptosis, endothelial inflammation and dysfunction under OGD/R conditions in vitro. In transgenic animal models, cardiac IRI similarly induced ERS and increased the expression of endothelial Ero1α, which was accompanied by vascular barrier impairment and myocardial inflammation. The above results demonstrated that Ero1α deletion could effectively abrogate the increase in ERS and attenuate endothelial damage both in vitro and in vivo, which was consistent with previous studies showing that limiting Ero1α content was essential for maintaining ER redox homeostasis and inhibiting ER stress.^19^, ^43^ Of note, when the allosteric disulphides are reduced, Ero1α can be activated and lead to ER hyperoxidation.^44^ In this study, whether OGD/R leads to endothelial ERS by activating Ero1α, and in this process, whether SIRT6 is involved in regulating its activity needed to be further investigated.

**FIGURE 7:**
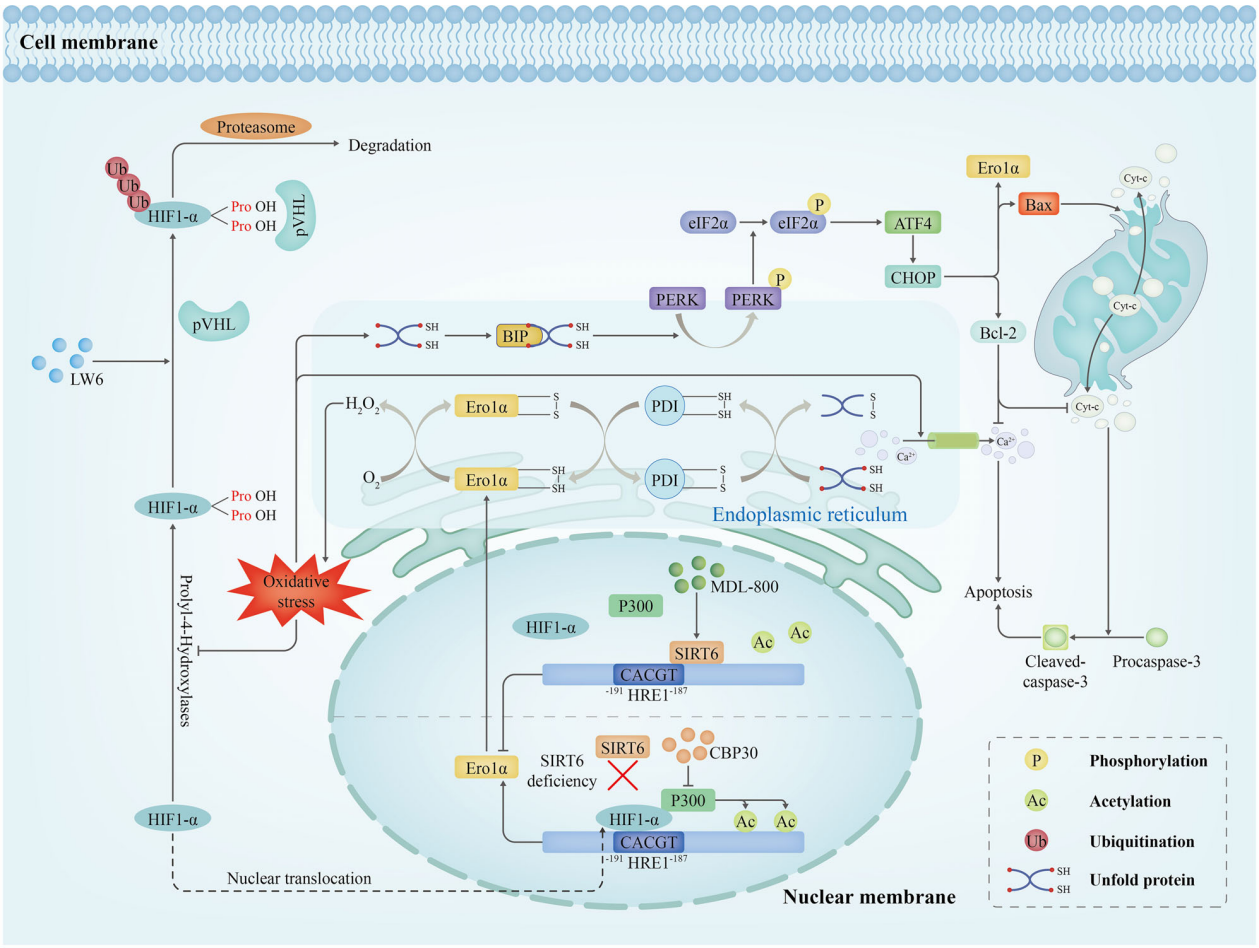
Diagram of the molecular mechanism of SIRT6‐Ero1α in I/R injury of cardiac microvascular endothelial cells. OGD/R‐induced oxidative stress promotes HIF1α accumulation and nuclear translocation to increase the expression of endoplasmic reticulum oxidase 1 alpha (Ero1α). Oxidative stress causes the accumulation of unfolded proteins in the ER, thereby initiating the unfolded protein response (UPR) to maintain ER homeostasis. In response to the UPR, Ero1α in the ER participates in protein folding, which inevitably results in the H_2_O_2_ accumulation. Finally, excessive oxidation and a persistent UPR in the ER occur. Over time, the persistent UPR could activate the PERK‐eif2α‐CHOP signaling pathway (a key endoplasmic reticulum stress [ERS] sensor) to lead to cell dysfunction and death. Subsequently, activation of the PERK‐eif2α‐CHOP signaling pathway also induces the expression of Ero1α to create a vicious cycle for further disruption of ER homeostasis. Throughout this process, SIRT6 serves as a repressor to inhibit the recruitment of HIF1α and p300 through deacetylating H3K9 at the Ero1α promoter, thereby suppressing the expression of Ero1α. In summary, we demostrated that SIRT6 attenuates the endothelial ischemia‒reperfusion injury through inhibiting Ero1α expression by promoting the chromatin eviction of HIF1α/p300, breaking the positive feedback loop between Ero1α and ERS.

In conclusion, the present study demonstrated that endothelial SIRT6 exerts a beneficial role in cardiac IRI by preserving cardiac microvascular function and integrity. At the molecular level, SIRT6 modulate endothelial functions by reducing ERS hyperactivation through inhibiting HIF1α/p300‐mediated Ero1α expression (Figure 7). More importantly, activation of the deacetylase activity of SIRT6 by MDL‐800 inhibited HIF1α occupation within Ero1α promoter and downregulated Ero1α expression. Therefore, data from this study showed the potential benefits of pharmacological activation of SIRT6 and set a stage for further investigating the endothelial SIRT6 as a novel therapeutic target in treating cardiac IRI.

## 4 MATERIALS AND METHODS

### 4.1 Cell culture and treatments

HEK 293T cells were obtained from ATCC, and cultured in Dulbecco's modified Eagle's medium (DMEM) containing 10% foetal bovine serum (FBS) and 1% antibiotics (penicillin and streptomycin) in 5% CO_2_ incubator at 37°C. SIRT6‐knockout (SIRT6‐KO) 293T cells were constructed by Hanbio Biotechnology (Shanghai, China) using the CRISPR/Cas9 system. The SIRT6 gRNA sequence was 5ʹ‐GTACGTCCGAGACACAGTCG‐3ʹ. Knockdown efficiency was measured by Western blotting.

Primary human umbilical endothelial cells (HUVECs) were cultured in endothelial cell medium (ECM) containing 5% FBS, 1% antibiotics (penicillin and streptomycin) and 1% endothelial cell growth supplement (ECGS) in 5% CO_2_ incubator at 37°C.

To establish endothelial cell model of oxygen–glucose deprivation/reperfusion (OGD/R) injury in vitro, HUVECs were cultured in glucose‐free DMEM (Gibco) and placed under hypoxic conditions (1% O_2_, 94% N_2_, 5% CO_2_) at 37°C for the specified time, and then moved to normal medium under normoxic condition.

### 4.2 Viral transfection

Adenoviruses encoding human shSIRT6 (Ad‐shSIRT6) and shEro1α (Ad‐shEro1α) and their respective negative controls were constructed by Hanbio Biotechnology (Shanghai, China). The shSIRT6 sequence was 5ʹ‐GCTACGTTGACGAGGTCATGA‐3ʹ; the shEro1α sequence was GGGCTTTATCCAAAGTGTTACCATT. According to the manufacturer's instructions and previous reports,^45^ HUVECs were transfected with Ad‐shSIRT6 and/or Ad‐shEro1α and the corresponding Ad‐Ctrl at multiplicities of infection (MOI) of 100. The transfection efficiency was determined by Western blotting.

Recombinant pGLV3 lentiviral vectors harbouring SIRT6‐WT or different mutants (H133Y, G60A and R65A) were purchased from Hanbio Biotechnology (Shanghai, China). To establish SIRT6‐ and mutant‐overexpressing cells in vitro, the lentiviral vectors were transfected into SIRT6‐KO 293T cells. Briefly, SIRT6‐KO 293T cells were cultured in cell culture dish for reaching 60% confluence, then the cells were individually transfected with the virus described above. After 12 h of transfection, the cells were continued to be cultured in fresh DMEM containing 10% FBS.

Mouse adeno‐associated virus‐9 expressing shEro1α (AAV9‐shEro1α) or Sirt6 (AAV9‐Sirt6) genome particles were obtained from Hanbio Biotechnology (Shanghai, China). The mouse shEro1α sequence was GGACCAAGTTATGAGTTCCAGCTAA. One hundred microliters of AAV9‐shEro1a or AAV9‐Sirt6 particles or the negative control (AAV‐Ctrl) at a density of 5  ×  10^12^ v.g./ml were injected into 4‐week‐old WT mice or ecSirt6^−/−^ mice via the caudal vein, respectively. Four weeks later, mouse CMECs were isolated to measure the Ero1a‐knockdown and Sirt6‐overexpression efficiency by Western blotting.

### 4.3 Western blotting

The detailed procedures for the preparation of cellular lysates and the Western blot analysis have been described previously.^15^ The primary antibodies used in this study were as follows: HIF1α (36169S, CST, USA), Ero1α (3264S, CST, USA; 702709, Thermo Fisher, USA), p‐PERK (PA5‐102853, Thermo Fisher, USA), PERK (PA5‐120620, Thermo Fisher, USA), BIP (ab21685, Abcam, USA), p‐eif2α (3398S, CST, USA), eif2α (5324S, CST, USA), CHOP (2895S, CST, USA), CC3 (19677‐1‐AP, Proteintech, USA), Bax (ab32503, Abcam, USA), Bcl‐2(ab32124, Abcam, USA), SIRT6 (ab191385, Abcam, USA), p‐p65 (ab76302, USA), p65 (ab32536, USA), ICAM‐1 (ab53013, ab171123, Abcam, USA), VCAM‐1 (ab134047, Abcam, USA), VE‐cadherin (ab33168, Abcam, USA) and β‐actin (ab8226, Abcam, USA) was applied as the internal standard substances.

### 4.4 RNA extraction and quantitative real‐time PCR (qPCR)

Total RNA extracted from cells using TriZol Total RNA Isolation Kit (Sangon Biotech, China) were reverse transcribed into cDNA using Prime Script RT Master Mix (Takara Biotechnology, Japan). Then, qPCR was performed on CFX Connected^™^ Real‐Time PCR Detection System (Bio‐Rad, USA), using Maxima SYBR Green/Rox qPCR Master Mix (Thermo Fisher Scientific). Fold changes in gene expression in the samples was normalized to β‐actin and calculated by the ΔΔCT method (2^−∆∆Ct^). The following primers was used: Ero1α forward: GCCAGGTTAGTGGTTACTTGG; reverse: GGCCTCTTCAGGTTTACCTTGT; β‐actin forward: TGGTATCGTGGAAGGACTC; reverse: AGTAGAGGCAGGGATGATG.

### 4.5 Mitochondrial ROS detection

For the measurement of mitochondrial ROS production, cells were assessed through using MitoSOX^™^ reagent (M36008, Thermo Fisher Scientific, USA) under several circumstances.^15^ Briefly, the indicator was dissolved in DMSO, and diluted to a final concentration of 5 μmol/L with serum‐free medium, and then incubated with treated cells at 37°C for 15 min. The cells were washed three times with warm PBS buffer and imaged under epifluorescence microscopy.

### 4.6 Flow cytometric analysis of apoptosis

Cell apoptosis under several circumstances was assessed by using Annexin V‐FITC/PI double staining (556547, BD Pharmingen, USA).^46^ Cells were harvested and washed two times with ice‐cold PBS, and then resuspended in 400 μL of 1× binding buffer at a concentration of 1 ×× 10^6^ cells/mL. Subsequently, the cell suspension was dyed for 15 min with 5 μL Annexin V‐FITC or/and 2 μL PI solutions at room temperature (RT) in the dark, and subjected to flow cytometric analysis.

### 4.7 Cell monolayer permeability assay

The in vitro endothelial monolayer permeability assay was described previously.^18^ Briefly, HUVECs were cultured in the upper chamber transwell inserts (0.4 μm) with ECM for reaching appropriate confluence and then exposed to OGD/R under several circumstances. Then, the normal ECM was removed, and the upper chamber added with fresh ECM containing 1 mg/mL FITC‐dextran (46945, Merck, German), and the lower chamber added with medium without phenol red (21063029, Thermo Fisher, USA). After 1 h of free permeation, 100 μL of medium in the upper and lower chamber was collected, and the fluorescence intensity was quantified by a fluorescence plate reader.

### 4.8 Immunofluorescence staining

Frozen sections (6 μm) of mouse heart tissue were fixed in ice‐cold acetone at −20°C for 10 min, and then blocked by 5% bovine serum albumin (BSA) for 1 h at RT. After being washed four times with PBS, the sections were incubated with VE‐cadherin (ab33168, Abcam, USA) and CD31 (3528S, CST, USA) primary antibodies overnight at 4°C. Frozen sections were then washed four times with PBS and stained with FITC‐labelled secondary antibodies for 1 h at RT. Finally, the sections were incubated with DAPI for 15 min at 4°C, and imaged with confocal microscopy.

### 4.9 Chromatin immunoprecipitation

ChIP Kit (ab500, Abcam, USA) was used to detect the levels of H3K9Ac, HIF1α and p300 at Ero1α promoter under several circumstances.^47^ Cells were crosslinked and lysed, followed by sonication to shear the DNA to fragments on ice. The fragments were then immunoprecipitated with specific antibodies against H3K9ac, HIF1α and p300, and normal rabbit IgG as a negative control. After a series of elution and decrosslinking steps, the decrosslinked DNA was extracted for PCR analysis with primers.

### 4.10 Generation of endothelial‐specific Sirt6‐knockout mice

Endothelial‐specific Sirt6‐knockout (ecSirt6^−/−^) mice (on a C57/Bl6J background) were developed by Cyagen Biosciences lnc. and litter of wild‐type (WT) mice served as control. Genotyping by tail preparation and PCR were performed at 2 weeks of age. All animal procedures were conducted under the guidelines of animal welfare and were approved by the Animal Ethics Committee of Zhongshan Hospital, Fudan University.

### 4.11 Cardiac ischemia–reperfusion injury surgery

Mouse cardiac ischemia–reperfusion surgery was performed as previously described.^48^ Briefly, 2% isoflurane was used to anesthetize male mice in 100% O_2_ ventilation at 2 L/min. Following left thoracotomy to expose the heart, the left anterior descending (LAD) coronary artery was obstructed by ligation using a 6.0 silk suture for 45 min to induce cardiac ischemia. Then, the slipknot was released to allow myocardial reperfusion for 6 h. Left‐side thoracotomy and pericardial exposure was performed in the sham‐operated mice, leaving the LAD intact.

### 4.12 Measurement of infarct area and area at risk

Infarct size determination was performed as previously described.^49^ After reperfusion, the LAD was occluded with a 6.0 silk suture at the ligation site. The 1% Evans blue dye was perfused into the ascending aorta and coronary arteries to distinguish the myocardial ischemia area at risk (AAR). Then the hearts were excised and washed with PBS until the remaining blood in the heart tissue is completely removed, followed by being frozen at −20°C for 1 h. The frozen hearts were then cross‐sectioned at a thickness of 1 mm and incubated in PBS (pH 7.4) containing 1% 2,3,5‐triphenyltetrazolium chloride (TTC) for 15 min at 37°C in the dark to distinguish the infarct area (IA). Subsequently, slices were washed with PBS and then fixed with 4% paraformaldehyde (PFA) overnight at 4°C . The IA (pale), the AAR (red), and the total left ventricular (LV) area were analysed using Image‐Pro Plus 6.0 (NIH, Bethesda, MD).

### 4.13 Histological analysis

Serial cryosections of myocardial tissues were prepared with a thickness of 5 μm for histological analysis. The cryosections were then fixed in 4% PFA and washed three times with PBS for 10 min each time. Then, plasma membranes were permeabilized by using Triton X‐100. Then the heart sections were incubated in 5% BSA for 1 h at RT. Indicated primary antibodies (1:200) were added and incubated overnight at 4°C, followed by incubation with secondary antibodies for 1 h at RT. Finally, the stained slides were imaged with an Olympus IX71 or Zeiss Pascal confocal microscope.

### 4.14 Echocardiography

To evaluate cardiac function in vivo, transthoracic echocardiography (VisualSonics VeVo 2100 Imaging System, Toronto, Canada) was performed on isoflurane‐anesthetized mice.^48^ At the target heart rate (400–550 bpm), the LV ejection fraction (LVEF), fractional shortening (FS) and other indices of systolic function were calculated from M‐mode echocardiograms. At the end of imaging procedure, all mice fully recovered from anaesthesia.

### 4.15 Statistical analysis

Data from this study are presented as the mean ± standard deviation (SD) of three or six independent experiments. The statistical significance of the difference between different groups was determined by two‐tailed unpaired Student's *t*‐test or one‐way ANOVA, as applicable. *p* < .05 was considered statistically significant (*) while *p* > 0.05 was considered not significant (ns).

## CONFLICT OF INTEREST STATEMENT

The authors declare no conflicts of interest.

## Supporting information

Supplementary Figure 1 (A‐B) The change in the protein level of Ero1α at different time points of OGD (A) and reperfusion after OGD for 12 h (B) was determined by Western blotting. (C) The efficiency of adenovirus‐mediated overexpression and knockdown of SIRT6 in HUVECs was determined by Western Blotting. (D) Schematic diagram of the transwell permeability model. One‐way ANOVA followed by post hoc Tukey's test for A and B, and two‐tailed unpaired Student's t‐test for C. ns means no significance; * means p < 0.05.

Supplementary Figure 2 (A) The efficiency of adenovirus‐mediated knockdown of Ero1α in HUVECs was determined by Western Blotting. (B) The efficiency of SIRT6 knockout in 293T cells was determined by Western blotting. (C) ChIP analysis of HIF1α occupancy on the Ero1α promoter was performed with IgG or anti‐HIF1α antibodies in HUVECs with OGD/R treatment. WT indicates the control of SIRT6 knockout 293T cells, and KO indicates the SIRT6 knockout 293T cells; Ad‐shEro1α indicates the AAV mediated Ero1α knockdown cells and Ad‐shCtrl indicates the corresponding controls. Two‐tailed unpaired Student's t‐test for A and One‐way ANOVA followed by post hoc Tukey's test was used for C. ns means no significance; * means p < 0.05.

Supplementary Figure 3 (A) Schematic model illustrating the generation of conditional knockout mice in which Sirt6 was specifically ablated in endothelial cells via by using Cre‐LoxP recombination system. Exons 3, 4, 5 and 6 were deleted on Cdh5‐Cre‐mediated recombination. (B) The protein expression of Sirt6 in mouse CMECUs was detected by Western blotting. (C) The mice were confirmed by tail genotyping at 2 weeks of age. (D) The protein expression of Sirt6 was detected in CMECs of ecSirt6^−/−^ mice after 4 weeks of adeno‐associated virus injection by Western blotting. (E) Representative flow cytometry images showing the CD31 expression in isolated mouse coronary endothelial cells. (F) Representative immunofluorescence images showing CD31 expression in cultured mouse coronary endothelial cells. Scale bars = 40 μm. (G) Statistical analysis of Figure 5E.

Supplementary Figure 4 (A) Representative cardiac M‐mode echocardiograms and cardiac functions (LVEF, LVFS) were quantified. N = 6/group. (B) Co‐staining of VE‐Cadherin and CD31 was performed to characterize endothelial barrier integrity. Scale bars = 50 μm. N = 6/group. (C) Representative images showing the Ly6G^+^ neutrophil infiltration in myocardial tissues. Scale bars = 100 μm or 50 μm (enlarged groups). N = 6/group. (D) The infarcted area was quantified by TTC staining. N = 6/group. (E) ERS markers were detected by Western blotting in isolated mouse CMECs after cardiac IR surgery. N = 6/group. Two‐tailed Student's unpaired t test for A, Dand E. ns means no significance; * means p < 0.05

Supplementary Figure 5 (A) The experimental protocol of mice subjected to IRI in vivo. AAV9‐Ero1α shRNA particles or negative control were injected into 4‐week‐old ecSirt6^−/−^ mice or WT mice via the tail vein. Four weeks after transfection, the mice underwent cardiac IR according to the experimental design. (B‐C) The knockdown efficiency of endothelial (B) and myocardial (C) Ero1α was detected by Western blotting.

Supplementary Figure 6 (A) Mitochondrial superoxide levels in mouse CMECs after cardiac IR surgery were determined via using mitochondrial superoxide indicators. N = 6/group. (B) Statistical analysis of Figure 6E. (C) Western blotting was used to detect the activation of p65 and inflammation in mouse CMECs after cardiac IR. N = 6/group. One‐way ANOVA followed by post hoc Tukey's test was used. ns means no significance; * means p < 0.05.
