# Supplementary figures and images for: SIRT6 deficiency in endothelial cells exacerbates oxidative stress by enhancing HIF1α accumulation and H3K9 acetylation at the Ero1α promoter
Source: Clin Transl Med. 2023 Aug 20;13(8):e1377. doi: 10.1002/ctm2.1377 (PMC10440057; doi:10.1002/ctm2.1377)

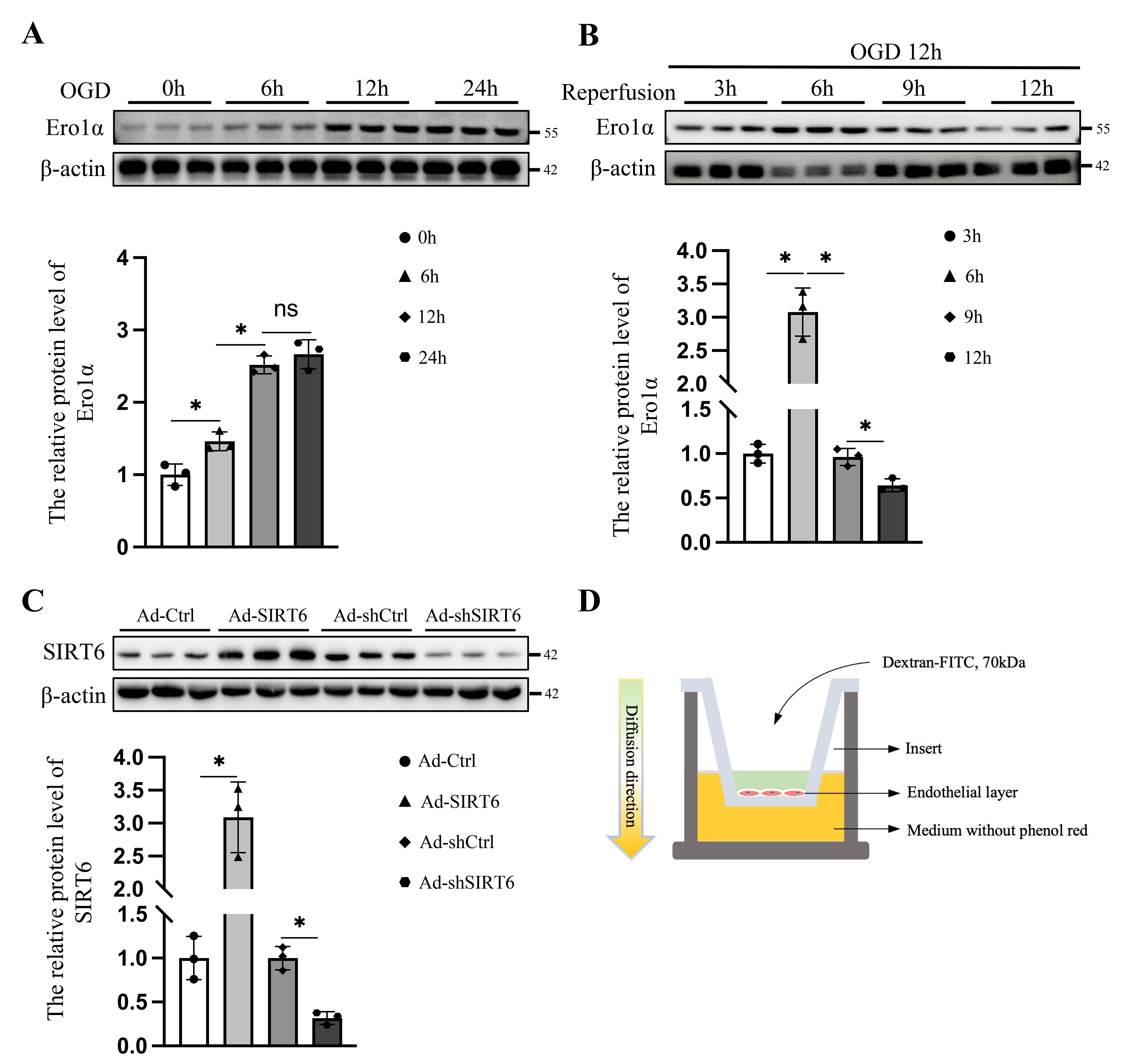

Supplement: Supplementary file 1 — Supplementary Figure 1 (A‐B) The change in the protein level of Ero1α at different time points of OGD (A) and reperfusion after OGD for 12 h (B) was determined by Western blotting. (C) The efficiency of adenovirus‐mediated overexpression and knockdown of SIRT6 in HUVECs was determined by Western Blotting. (D) Schematic diagram of the transwell permeability model. One‐way ANOVA followed by post hoc Tukey's test for A and B, and two‐tailed unpaired Student's t‐test for C. ns means no significance; * means p < 0.05. [file CTM2-13-e1377-s006.jpg]

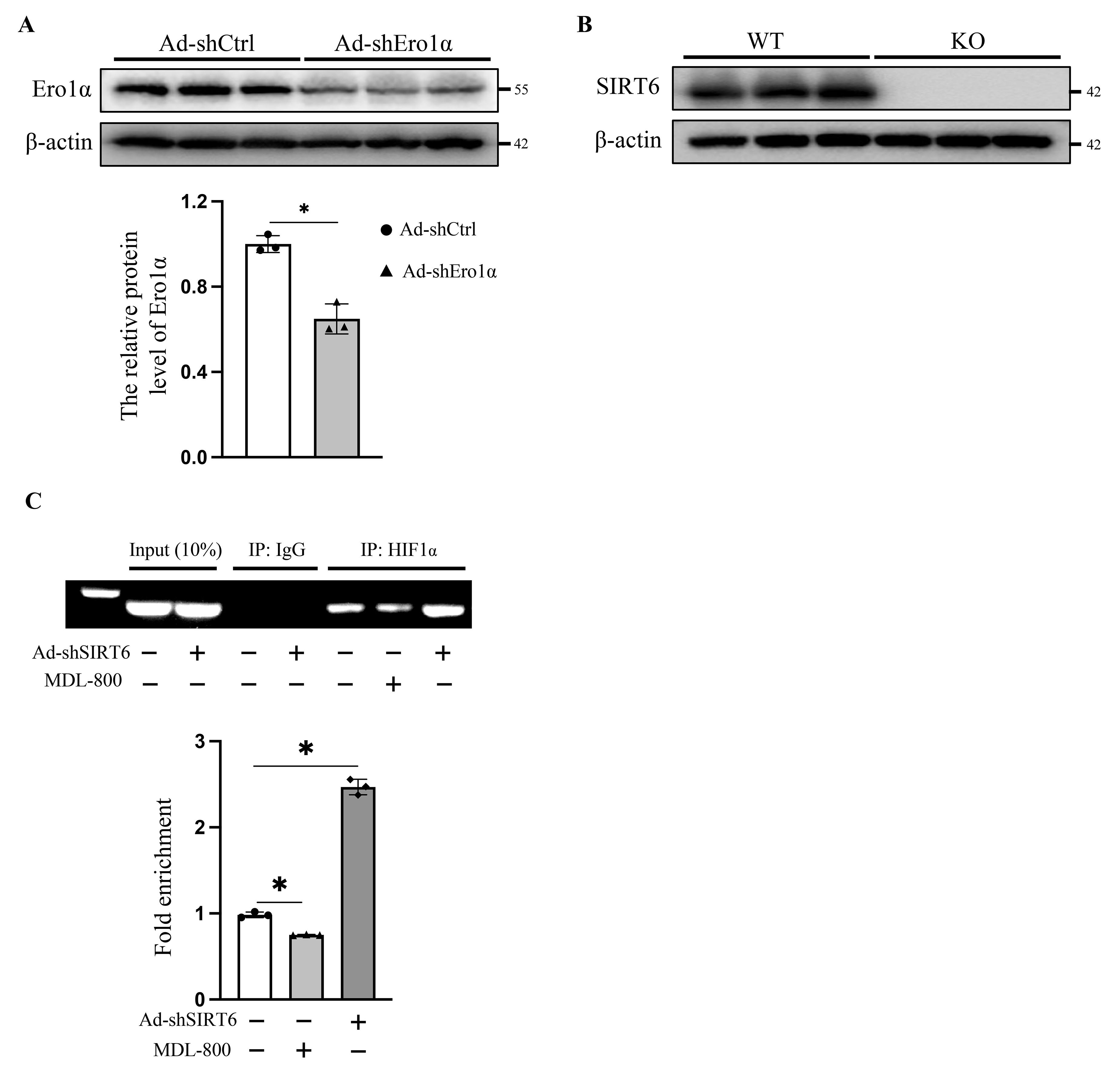

Supplement: Supplementary file 2 — Supplementary Figure 2 (A) The efficiency of adenovirus‐mediated knockdown of Ero1α in HUVECs was determined by Western Blotting. (B) The efficiency of SIRT6 knockout in 293T cells was determined by Western blotting. (C) ChIP analysis of HIF1α occupancy on the Ero1α promoter was performed with IgG or anti‐HIF1α antibodies in HUVECs with OGD/R treatment. WT indicates the control of SIRT6 knockout 293T cells, and KO indicates the SIRT6 knockout 293T cells; Ad‐shEro1α indicates the AAV mediated Ero1α knockdown cells and Ad‐shCtrl indicates the corresponding controls. Two‐tailed unpaired Student's t‐test for A and One‐way ANOVA followed by post hoc Tukey's test was used for C. ns means no significance; * means p < 0.05. [file CTM2-13-e1377-s001.jpg]

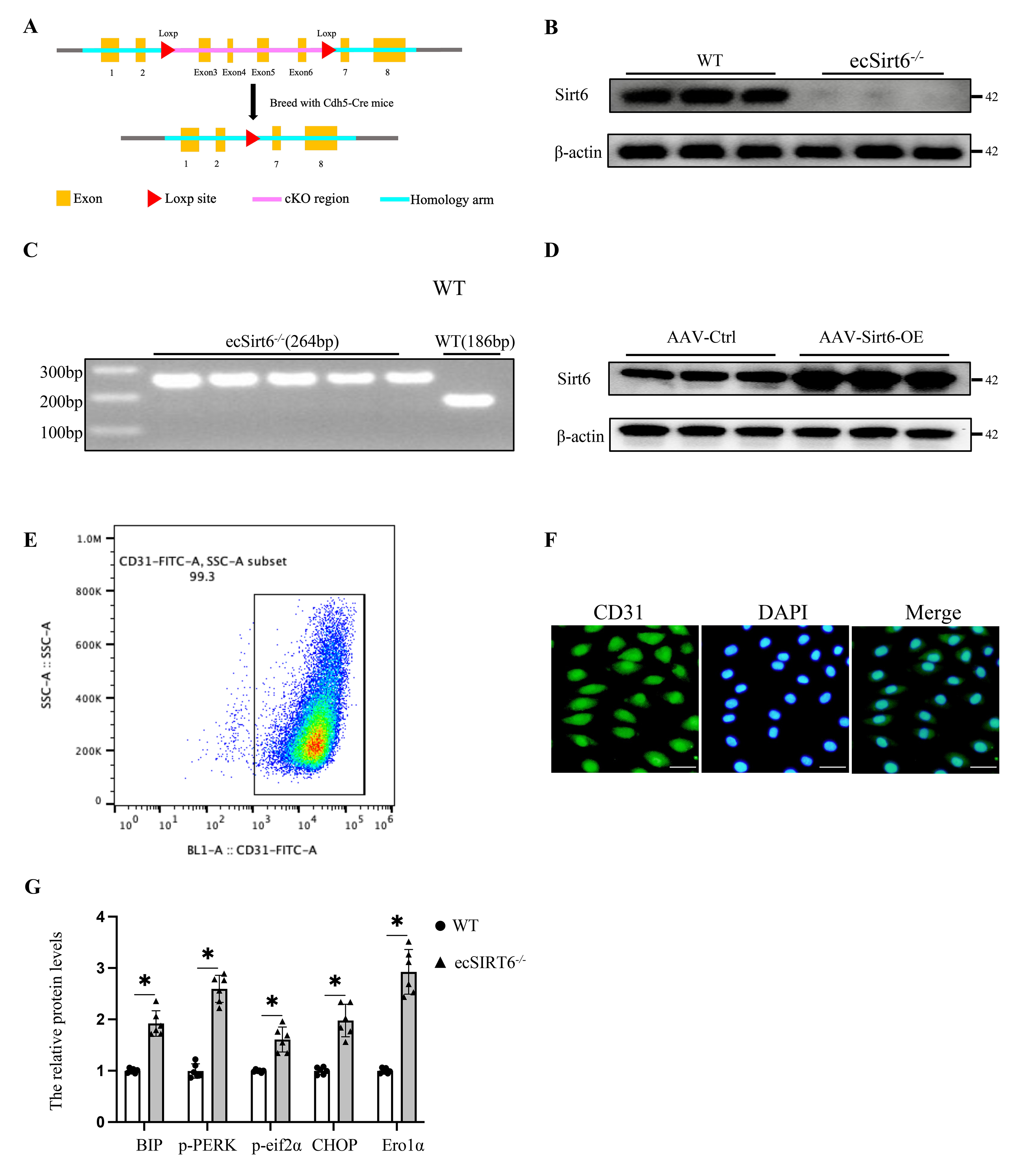

Supplement: Supplementary file 3 — Supplementary Figure 3 (A) Schematic model illustrating the generation of conditional knockout mice in which Sirt6 was specifically ablated in endothelial cells via by using Cre‐LoxP recombination system. Exons 3, 4, 5 and 6 were deleted on Cdh5‐Cre‐mediated recombination. (B) The protein expression of Sirt6 in mouse CMECUs was detected by Western blotting. (C) The mice were confirmed by tail genotyping at 2 weeks of age. (D) The protein expression of Sirt6 was detected in CMECs of ecSirt6−/− mice after 4 weeks of adeno‐associated virus injection by Western blotting. (E) Representative flow cytometry images showing the CD31 expression in isolated mouse coronary endothelial cells. (F) Representative immunofluorescence images showing CD31 expression in cultured mouse coronary endothelial cells. Scale bars = 40 μm. (G) Statistical analysis of Figure 5E. [file CTM2-13-e1377-s003.jpg]

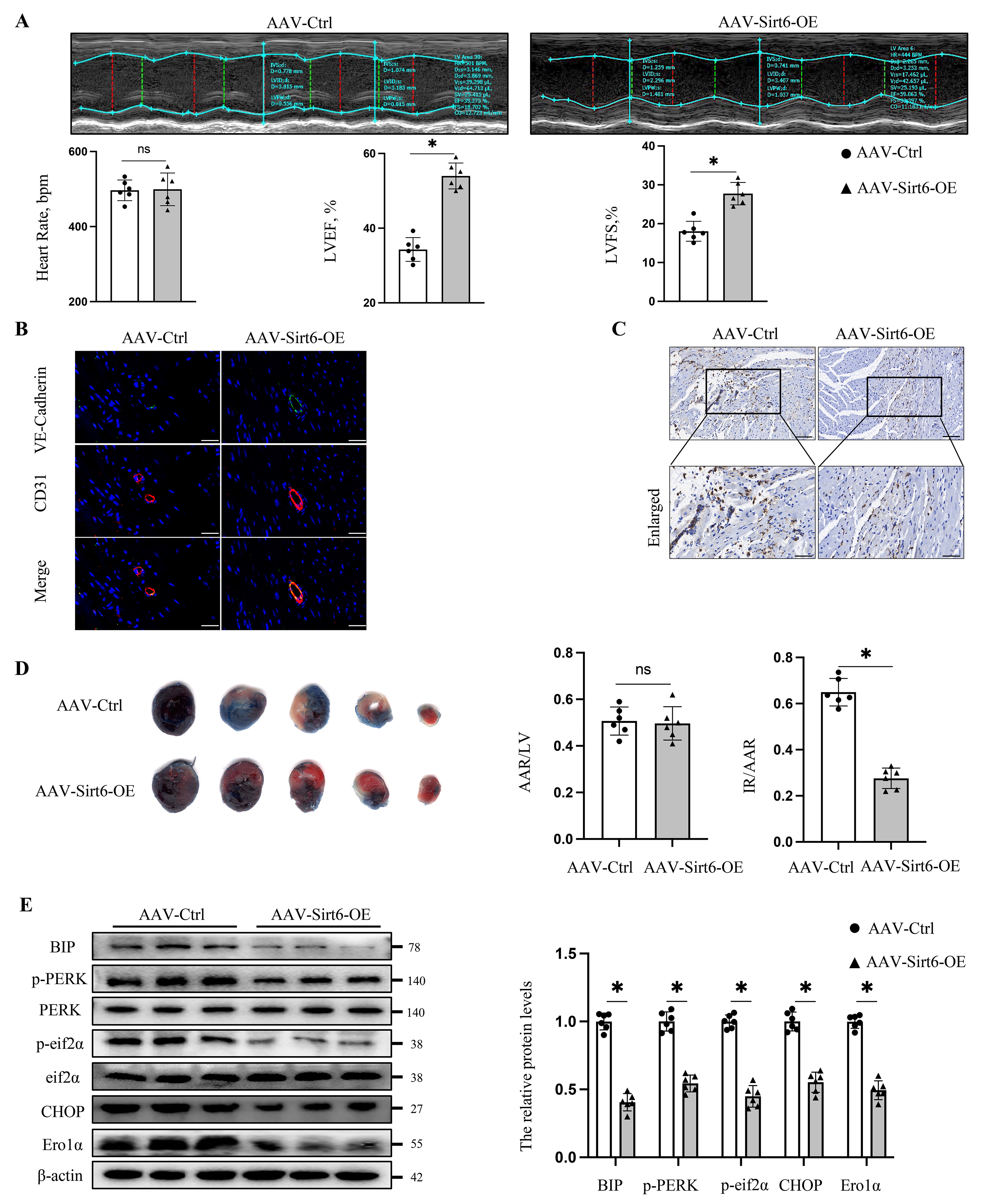

Supplement: Supplementary file 4 — Supplementary Figure 4 (A) Representative cardiac M‐mode echocardiograms and cardiac functions (LVEF, LVFS) were quantified. N = 6/group. (B) Co‐staining of VE‐Cadherin and CD31 was performed to characterize endothelial barrier integrity. Scale bars = 50 μm. N = 6/group. (C) Representative images showing the Ly6G+ neutrophil infiltration in myocardial tissues. Scale bars = 100 μm or 50 μm (enlarged groups). N = 6/group. (D) The infarcted area was quantified by TTC staining. N = 6/group. (E) ERS markers were detected by Western blotting in isolated mouse CMECs after cardiac IR surgery. N = 6/group. Two‐tailed Student's unpaired t test for A, Dand E. ns means no significance; * means p < 0.05 [file CTM2-13-e1377-s002.jpg]

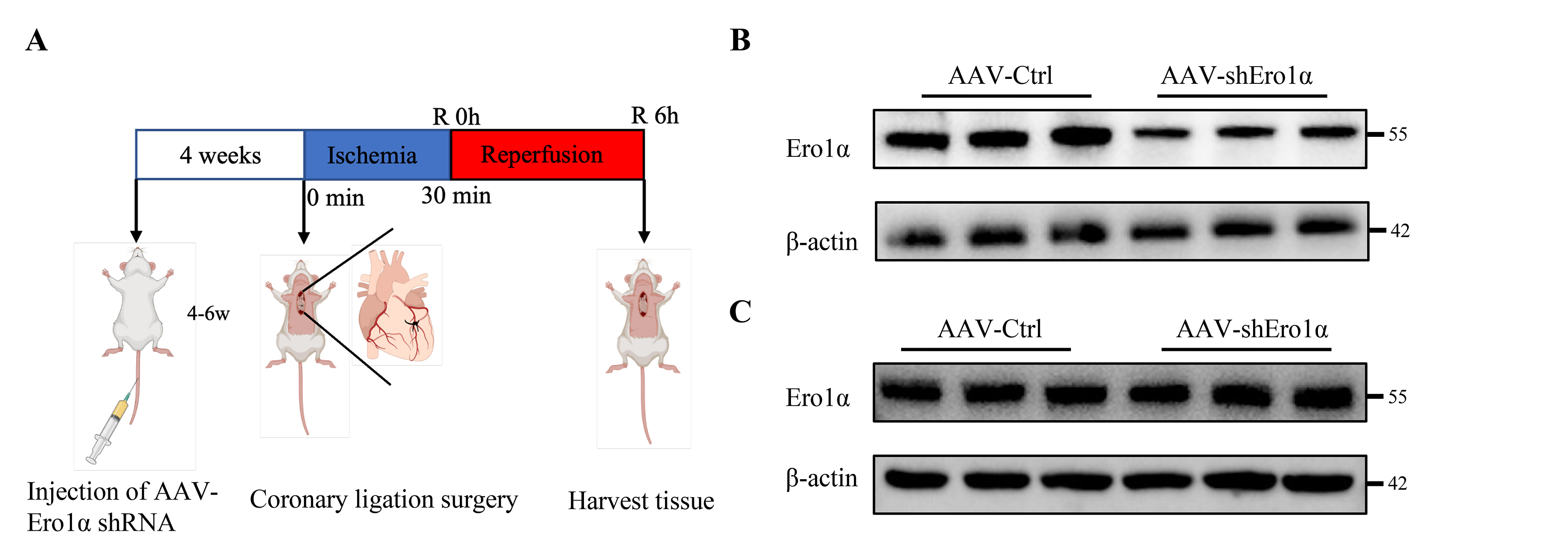

Supplement: Supplementary file 5 — Supplementary Figure 5 (A) The experimental protocol of mice subjected to IRI in vivo. AAV9‐Ero1α shRNA particles or negative control were injected into 4‐week‐old ecSirt6−/− mice or WT mice via the tail vein. Four weeks after transfection, the mice underwent cardiac IR according to the experimental design. (B‐C) The knockdown efficiency of endothelial (B) and myocardial (C) Ero1α was detected by Western blotting. [file CTM2-13-e1377-s005.jpg]

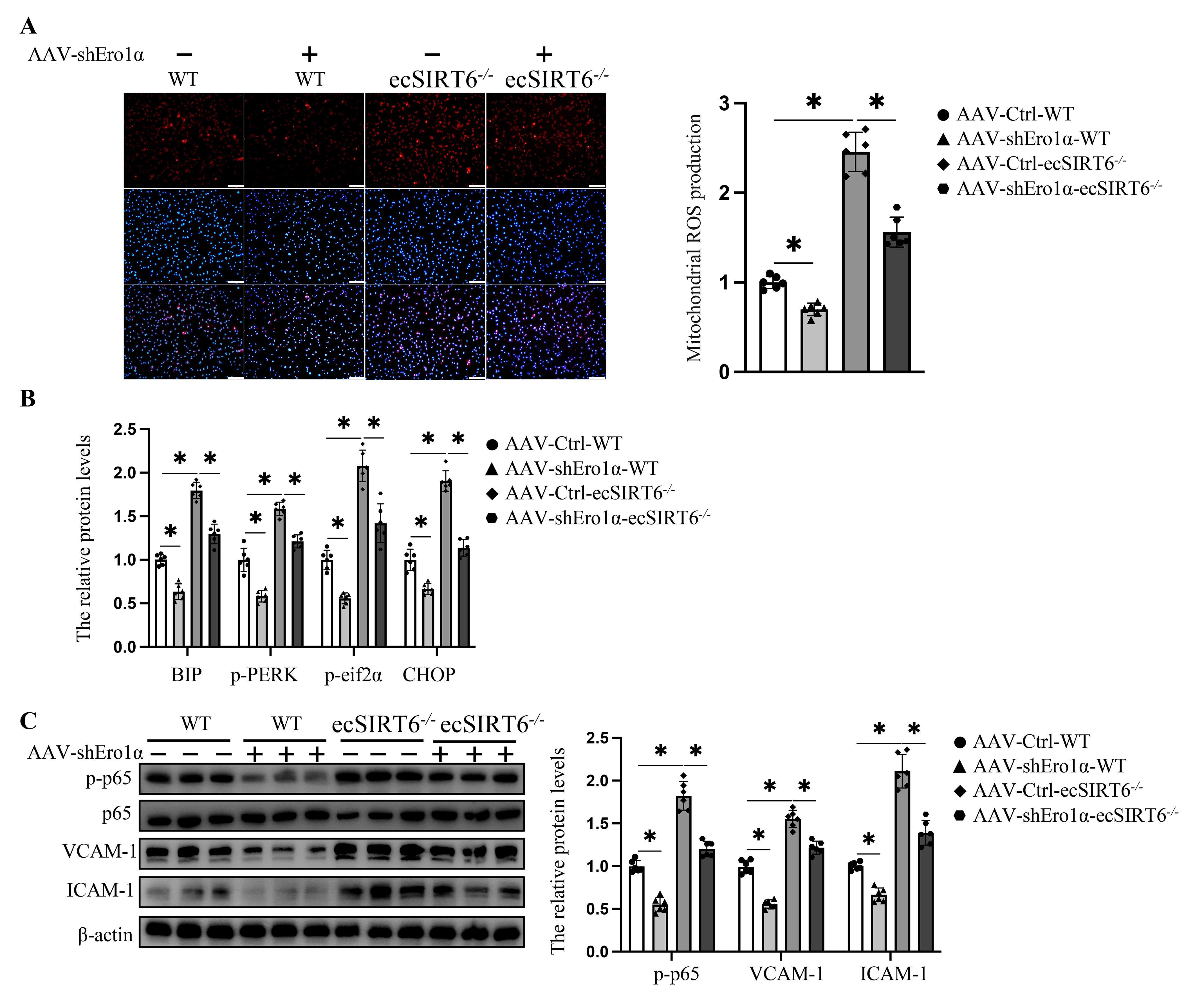

Supplement: Supplementary file 6 — Supplementary Figure 6 (A) Mitochondrial superoxide levels in mouse CMECs after cardiac IR surgery were determined via using mitochondrial superoxide indicators. N = 6/group. (B) Statistical analysis of Figure 6E. (C) Western blotting was used to detect the activation of p65 and inflammation in mouse CMECs after cardiac IR. N = 6/group. One‐way ANOVA followed by post hoc Tukey's test was used. ns means no significance; * means p < 0.05. [file CTM2-13-e1377-s004.jpg]
